# Phenotypic and Molecular Characterization of *Klebsiella pneumoniae* Species Complex Isolates from Clinically Healthy Poultry, with Identification of Novel *K. quasipneumoniae* ST9812

**DOI:** 10.3390/microorganisms14102138

**Published:** 2026-09-23

**Authors:** Nurdan Karacan Sever, Özlem Şahan Yapıcıer, Seyyide Sarıçam İnce

**Affiliations:** 1 Faculty of Veterinary Medicine, Department of Microbiology, Dicle University, TR-21200 Diyarbakır, Türkiye; 2 Bacteriology Diagnostic Laboratory, Republic of Turkey Ministry of Agriculture and Forestry Veterinary Control, Central Research Institute, TR-06020 Ankara, Türkiye; ozlem-sahan@hotmail.com; 3 Faculty of Veterinary Medicine, Department of Microbiology, Ankara University, TR-06110 Ankara, Türkiye; s.saricam-92@hotmail.com

**Keywords:** *Klebsiella pneumoniae* species complex, poultry, antimicrobial resistance, virulence-associated genes, multilocus sequence typing, *Klebsiella quasipneumoniae*, One Health

## Abstract

This cross-sectional, culture-based study aimed to recover *Klebsiella pneumoniae* species complex (KpSC) isolates from fecal samples collected from clinically healthy chicken and turkey flocks in Diyarbakır, Türkiye, and to characterize the sequence-confirmed isolates. A total of 419 fecal samples from small-scale, family-operated farms were examined. Presumptive isolates were screened by MALDI-TOF MS and confirmed by 16S rRNA and *rpoB* gene sequencing. Antimicrobial susceptibility testing, phenotypic ESBL and carbapenemase assays, targeted PCR detection of resistance- and virulence-associated genes, multilocus sequence typing (MLST), and phylogenetic analyses were performed. Six KpSC isolates were confirmed from the 419 samples examined (6/419, 1.43%): *K. pneumoniae* (n = 4), *K. quasipneumoniae* (n = 1), and *K. variicola* (n = 1). All six were resistant to ampicillin, amoxicillin, and enrofloxacin; two were multidrug-resistant, and one showed an ESBL phenotype. No carbapenemase phenotype or targeted carbapenemase genes were detected. *intI*1 and *bla*_SHV_ were each detected in four isolates, while *wab*G and *ent*B were present in all isolates. MLST identified six distinct sequence types, one of which, *K. quasipneumoniae* ST9812, was novel and associated with the novel *rpo*B allele 593, while one turkey-origin *K. pneumoniae* isolate belonged to ST15. These findings demonstrate phenotypic and molecular diversity among poultry-origin KpSC isolates and provide a basis for broader genomic investigation of their epidemiological significance.

## 1. Introduction

*Klebsiella pneumoniae* is a Gram-negative, nonmotile, facultatively anaerobic member of the *Enterobacteriaceae* and an opportunistic pathogen [1,2,3]. Molecular studies have demonstrated that organisms historically identified as *K. pneumoniae* comprise the *Klebsiella pneumoniae* species complex (KpSC) [4,5], which includes closely related species such as *K. pneumoniae*, *K. quasipneumoniae*, and *K. variicola* [6,7]. KpSC members can be found in environmental sources such as water, soil, and plants, as well as in the gastrointestinal and respiratory tracts of humans and animals, including food-producing animals [4,8,9]. Due to its antimicrobial resistance capacity, *K. pneumoniae* is an important member of the ESKAPE pathogen group [2,10] and is a major cause of pneumonia, urinary tract infections, and hospital-acquired infections in humans [3,4,11]. In poultry, *Klebsiella* spp. have been associated with respiratory infections and have also been isolated from diseased birds and poultry production environments [12,13].

*K. pneumoniae* can acquire and disseminate antimicrobial resistance determinants, including β-lactamase and carbapenemase genes, through mobile genetic elements [4,11]. Antimicrobial-resistant KpSC isolates have also been reported in poultry and poultry production environments, raising concerns about the circulation of resistant bacteria and resistance determinants within the food chain [4,11]. These findings support the investigation of poultry-associated KpSC within a One Health framework.

Multidrug-resistant *K. pneumoniae* isolates have been reported in poultry populations from different geographical settings [14,15,16]. Poultry-associated KpSC isolates may carry clinically relevant resistance determinants, including β-lactamase genes such as *bla*_CTX-M_, *bla*_SHV,_ and *bla*_TEM_ [4,11], plasmid-mediated quinolone resistance determinants such as *qnr*B and *qnr*S [14,17], and carbapenemase genes, including *bla*_NDM,_ *bla*_OXA-48,_ and *bla*_KPC_ [11,15,16]. These findings demonstrate the diversity of antimicrobial resistance mechanisms among poultry-origin isolates and support their continued molecular characterization.

The pathogenic potential of KpSC is influenced by virulence-associated traits involved in capsule and lipopolysaccharide biosynthesis, adhesion, and iron acquisition [1,18,19], as well as metabolic adaptation [20]. Virulence-associated genes have been reported in poultry- and livestock-origin isolates [19]. Characterizing these determinants together with antimicrobial resistance profiles can provide a broader description of the recovered isolates; however, gene detection does not establish expression or functional contribution.

Accurate species-level identification is important for characterizing KpSC diversity because conventional methods may not reliably distinguish closely related *Klebsiella* species. Although 16S rRNA gene sequencing is widely used for bacterial identification, it may also have limited discriminatory resolution within KpSC [21]. Multilocus sequence typing (MLST) provides a standardized approach for characterizing genetic diversity by assigning isolates to sequence types based on variation in seven housekeeping genes [19,22].

In Türkiye, poultry-associated antimicrobial resistance has previously been investigated in both intestinal and food chain settings. Kaya et al. [23] examined *E. coli* and *Klebsiella* recovered from chicken cecal contents using conventional identification and phenotypic antimicrobial susceptibility testing. Subsequent studies characterized ESBL-producing Enterobacterales, including *K. pneumoniae*, recovered from conventional and organic chicken meat [24]. More recently, Kürekci et al. [25] used whole-genome sequencing to characterize ESBL-producing *K. pneumoniae* from raw chicken meat, while Kürekci et al. [26] characterized tigecycline-resistant *Klebsiella* spp. recovered from chicken and turkey cecal samples and chicken meat. These studies demonstrate that antimicrobial-resistant *Klebsiella* has been documented in poultry and food animal sources in Türkiye. Nevertheless, data specifically addressing molecularly differentiated KpSC members recovered from fecal samples of clinically healthy poultry remain limited.

*K. pneumoniae* research has predominantly focused on human clinical isolates, while information on KpSC in animal and environmental settings remains limited [9,19]. Against this background, this cross-sectional, culture-based study aimed to recover and molecularly confirm KpSC isolates from fecal samples of clinically healthy chicken and turkey flocks on small-scale, family-operated farms in Diyarbakır and to characterize the confirmed isolates by molecular species identification, antimicrobial susceptibility testing, phenotypic ESBL and carbapenemase testing, targeted detection of resistance- and virulence-associated genes, and MLST. This integrated approach was intended to provide regional, isolate-level data for future surveillance and comparative genomic studies of poultry-associated KpSC.

## 2. Materials and Methods

### 2.1. Study Design and Sampling

In this cross-sectional study, a total of 419 fecal samples, comprising 351 chicken and 68 turkey samples, were collected between August 2024 and February 2025 from clinically healthy poultry flocks raised on small-scale, family-operated farms in Diyarbakır Province. Farms were selected by convenience sampling based on accessibility and the consent of the farm owners. No formal a priori sample size calculation was performed; the final number of samples collected was determined by the accessibility of consenting farms during the study period. Chicken samples were obtained from 32 farms located in the Çınar, Bağlar, and Central districts, whereas turkey samples were collected from two farms in the Central district. Freshly voided fecal material was collected from the floors of the poultry houses into sterile containers. The geographical distribution of the sampled flocks and the number of samples collected from each location are presented in Supplementary Table S1. Based on information obtained at the time of sampling, the sampled flocks had not received antimicrobial treatment during the preceding 10 days. No standardized biosecurity assessment was performed, and reliable historical antimicrobial use records and quantitative stocking density data were unavailable. Samples were transported to the laboratory under cold-chain conditions and processed for microbiological analysis.

### 2.2. Isolation and Identification of the Klebsiella pneumoniae Species Complex

For pre-enrichment, 5 g of each fecal sample was homogenized in 45 mL of buffered peptone water (BPW; Condalab, Madrid, Spain) and incubated aerobically at 37 °C for 18–24 h. Following pre-enrichment, a 10 µL loopful of each enrichment culture was inoculated separately onto MacConkey agar (Condalab, Madrid, Spain) and eosin methylene blue agar (EMB; Condalab, Madrid, Spain). The plates were incubated aerobically at 37 °C for 18–24 h. Pink, mucoid, and round colonies on MacConkey agar and pink or purple, mucoid, and shiny colonies on EMB agar were identified as presumptive isolates and purified [27]. When two morphologically distinct mucoid colony types were observed, both were subcultured for further identification.

Identification was conducted using a staged workflow. An initial group of 10 presumptive isolates was directly subjected to 16S rRNA and *rpo*B gene sequencing. The remaining presumptive isolates were subsequently screened using Matrix-Assisted Laser Desorption/Ionization Time-of-Flight Mass Spectrometry (MALDI-TOF MS, Bruker Daltonik GmbH, Bremen, Germany). Isolates identified as potential KpSC members by MALDI-TOF MS were subsequently subjected to 16S rRNA and *rpo*B gene sequencing using the same molecular identification procedure. When different colony codes represented the same isolate, they were considered laboratory duplicates and counted only once. Only unique isolates confirmed as KpSC members by sequence analysis were included in subsequent phenotypic and molecular characterization.

Amplification of the 16S rRNA gene was performed using PCR Master Mix (2×) (Thermo Fisher Scientific Baltics UAB, Vilnius, Lithuania) and the universal primers 27F and 1492R described by Lane [28] (Table S2). PCR cycling conditions were adapted from Sambrook and Russell [29]. The amplification consisted of an initial denaturation at 94 °C for 5 min followed by 30 cycles of denaturation at 94 °C for 1 min, annealing at 50 °C for 1 min, and extension at 72 °C for 1 min, with a final extension at 72 °C for 10 min. The *rpo*B gene was amplified using the primers listed within the MLST primer set in Supplementary Table S2 and under the PCR conditions described for the MLST analysis. *Klebsiella pneumoniae* ATCC 13883 was included as the positive control, and nuclease-free water was used as the negative control. Amplicons were examined by electrophoresis on 1.5% agarose gels and visualized under UV illumination.

Positive amplicons were purified enzymatically using ExoSAP-IT (Thermo Scientific, USA). Cycle sequencing was carried out using the identical primers and the BigDye Direct Cycle Sequencing Kit (Applied Biosystems, Carlsbad, CA, USA) according to the manufacturer’s instructions. Sequencing was performed on an ABI 3500 Genetic Analyzer (Applied Biosystems, USA). Raw sequence data were analyzed, and consensus sequences were generated using CLC Main Workbench v.8 (QIAGEN, Aarhus, Denmark). The obtained sequences were compared with reference sequences available in the GenBank database using the Basic Local Alignment Search Tool (BLASTn, https://blast.ncbi.nlm.nih.gov/Blast.cgi?PROGRAM, accessed on 1 April 2026). The validated 16S rRNA gene sequences were subsequently deposited in GenBank and assigned accession numbers (ANs).

### 2.3. Antimicrobial Susceptibility Testing

Antimicrobial susceptibility of the KpSC isolates was determined using the Kirby–Bauer disk diffusion method. Bacterial suspensions were adjusted to a 0.5 McFarland turbidity standard using sterile deionized water. The prepared inoculum was inoculated onto the surface of Mueller Hinton Agar (MHA; Condalab, Madrid, Spain). Twelve different antimicrobial disks (Bioanalyse, Ankara, Turkey) selected from six classes were placed onto the inoculated medium. The tested antimicrobials were as follows: amoxicillin (AX, 10 µg), ampicillin (AM, 10 ug), chloramphenicol (C, 30 µg), ciprofloxacin (CIP, 5 µg), doxycycline (DO, 30 µg), enrofloxacin (ENR, 5 µg), florfenicol (FFC, 30 µg), gentamicin (CN, 10 µg), kanamycin (K, 30 µg), nalidixic acid (NA, 30 µg), tetracycline (TE, 30 µg), and trimethoprim/sulfamethoxazole (1.25/23.75 µg). The inoculated plates were incubated aerobically at 37 °C for 18–24 h. The KpSC isolates were tested simultaneously in a single antimicrobial susceptibility testing run. *Escherichia coli* ATCC 25922 was included as the primary CLSI quality control strain, while *Klebsiella pneumoniae* ATCC 13883 was included as an additional reference strain. Following incubation, the inhibition zone diameters of the test and control strains were measured and recorded in the laboratory raw data records. The antimicrobial susceptibility results and quality control performance were evaluated according to the CLSI criteria [30].

### 2.4. Multidrug Resistance (MDR) and Multiple Antibiotic Resistance Index (MAR Index)

According to the standardized definitions proposed by Magiorakos et al. [31], MDR was defined as non-susceptibility to at least one antimicrobial agent in three or more antimicrobial categories. A similar criterion, based on resistance to three or more antimicrobial classes, has also been applied in a recent veterinary antimicrobial resistance study by Xiang et al. [32]. The multiple antibiotic resistance (MAR) index calculated for each isolate was obtained by dividing the number of antimicrobials to which the isolate was resistant by the total number of tested antimicrobials [33].

### 2.5. Phenotypic Analysis of Extended-Spectrum Beta Lactamase (ESBL) Activity

The ESBL production of the isolates was determined using the Combined Disk Test (CDT) method in accordance with the criteria specified in the CLSI guidelines [30]. In this context, ceftazidime (CAZ: 30 μg), cefotaxime (CTX: 30 μg) and cefpodoxime (CPD: 10 μg) disks were utilized both alone and combined with the beta lactamase inhibitor clavulanic acid (CLA: 10 μg) (CAZ/CLA, 30/10 μg; CTX/CLA, 30/10 μg; CPD/CLA, 10/10 μg) (Bioanalyse, Ankara, Turkey). Bacterial suspensions prepared according to the 0.5 McFarland standard were inoculated onto the surface of Mueller Hinton Agar (MHA) (Condalab, Madrid, Spain), after which the disks were placed, and the plates were incubated at 37 °C for 18–24 h. According to CLSI standards, an isolate was defined as an ESBL producer if the inhibition zone diameter of any combination was ≥5 mm larger than the zone diameter of the corresponding single antimicrobial disc [30].

### 2.6. Phenotypic Analysis of Carbapenemase Production

Carbapenemase production of the isolates was analyzed using the Modified Carbapenem Inactivation Method (mCIM) defined in the CLSI protocols [30]. Within the scope of the test procedure, a 1 μL loopful of a fresh culture of each KpSC isolate was homogenized in 2 mL of tryptic soy broth (TSB; Condalab, Madrid, Spain). A 10 μg meropenem disk (Bioanalyse, Ankara, Turkey) was added to the prepared suspension, and the mixture was incubated at 37 °C for 4 h (±15 min). At the end of the incubation period, the meropenem disk was removed from the TSB and placed onto an MHA plate previously inoculated with *E. coli* ATCC 25922 (adjusted to a 0.5 McFarland standard). The plates were incubated at 37 °C for 18–24 h. According to the CLSI criteria, inhibition zone diameters between 6–15 mm or the presence of colony growth within the zone were interpreted as positive, zones ≥19 mm were interpreted as negative, and values between 16–18 mm as indeterminate. Accordingly, the carbapenemase activity of the isolates was evaluated based on their ability to inactivate meropenem.

### 2.7. Genomic DNA Extraction

Genomic DNA was extracted from the KpSC isolates using a commercial genomic DNA purification kit (GeneJET; Thermo Fisher Scientific Baltics UAB, Vilnius, Lithuania). DNA quantity and purity were assessed spectrophotometrically before molecular analyses.

### 2.8. Genotypic Detection of Class 1 Integron (intI1), β-Lactamase Genes, PMQR, and Carbapenemase Genes

The isolates were analyzed by conventional PCR (T100TM, Bio-Rad, Singapore) for Class 1 integron (*intI*1), β-lactamase genes (*bla*_SHV_, *bla*_TEM_, *bla*_OXA-1_, and *bla*_CTX-M_), PMQR determinants (*qnr*A, *qnr*B, *qnr*S, and *qep*A), and carbapenemase genes (*bla*_IMP-1_, *bla*_KPC-gp_, *bla_N_*_DM-1_, *bla*_OXA-48_, *and bla*_VIM_). The target panel was selected to represent major acquired antimicrobial resistance determinants previously reported in KpSC and poultry-associated Enterobacterales, including Class 1 integrons as markers of mobile gene capture, common β-lactamase genes, PMQR determinants, and major carbapenemase gene groups [3,11,16]. Because a targeted PCR approach was employed, the panel was not intended to encompass all possible resistance mechanisms. Each 25 µL reaction mixture contained 2 µL of template DNA, 1 µL of each forward and reverse primer (10 pmol) (Sentebiolab, Ankara, Türkiye), 0.5 µL of dNTP mixture (10 mM), 3 µL of MgCl_2_ (25 mM), 2.5 µL of 10× PCR buffer, 0.2 µL of Taq DNA polymerase (Thermo Fisher Scientific Baltics UAB, Vilnius, Lithuania), and 14.8 µL of nuclease-free water. For each target gene, DNA previously confirmed to be positive for the corresponding determinant was included as a positive amplification control. Detailed source and strain identifiers for these control DNA samples were not available in the retained laboratory records and therefore could not be reported. Nuclease-free water was included as the no-template negative control in each PCR assay. The primer sequences for i*ntI*1, β-lactamase genes, PMQR determinants, and carbapenemase genes were obtained from the corresponding references listed in Supplementary Table S2. PCR amplification was performed using standardized cycling conditions adapted from Sambrook and Russell [29], with target-specific annealing temperatures as specified in Supplementary Table S2. For the carbapenemase genes, preliminary reactions were conducted at different annealing temperatures. The target-specific annealing temperatures corresponding to those reported in the respective primer references produced the clearest specific amplicons and were therefore used in the final assays (Table S2). Amplification consisted of an initial denaturation at 94 °C for 3 min followed by 30 cycles of denaturation at 94 °C for 60 s, target-specific annealing for 60 s, and extension at 72 °C for 60 s, with a final extension at 72 °C for 7 min. The primer sequences, final annealing temperatures, expected amplicon sizes, and corresponding references for all targets are provided in Supplementary Table S2. Electrophoretic separation of all amplicons was performed at 100 V for 60 min on 1.5% agarose gels containing SafeView™ Classic (Applied Biological Materials Inc., Richmond, VA, Canada). Documentation was carried out using a gel imaging system (Vilber Lourmat, Marne-la-Vallée, France).

### 2.9. Genotypic Detection of Virulence-Associated Genes

The investigated virulence-associated genes were selected to represent functional categories relevant to the colonization and pathogenic potential of KpSC, including lipopolysaccharide and capsular polysaccharide production, iron acquisition, and allantoin utilization. These targets were also selected because they have previously been investigated in poultry- or livestock-associated *Klebsiella* isolates [18,19,34]. Accordingly, the virulence-associated genes were categorized according to their biological functions: *wab*G for lipopolysaccharide biosynthesis; *rmp*A and *mag*A for capsular polysaccharide synthesis and regulation; *ent*B, *kfu*, *iro*N, and *iuc*A for iron acquisition; and *all*S for allantoin utilization. The genes were amplified using the same 25 µL PCR reaction mixture described for the antimicrobial resistance determinants. Previously confirmed target-positive DNA was included as the positive amplification control for each virulence-associated gene assay, as described for the antimicrobial resistance determinants. Nuclease-free water was included as the no-template negative control in each PCR assay. Preliminary reactions were conducted at different annealing temperatures, and the clearest specific amplification products were obtained at the target-specific temperatures reported by Hou et al. [19]; these temperatures were therefore used in the final assays. Thermal cycling consisted of an initial denaturation at 95 °C for 3 min followed by 30 cycles of denaturation at 94 °C for 25 s, target-specific annealing (Table S2) for 25 s, and extension at 72 °C for 1 min, with a final extension at 72 °C for 5 min. The primer sequences, target-specific annealing temperatures, expected amplicon sizes, and corresponding references are provided in Supplementary Table S2. Amplicons were separated and visualized by agarose gel electrophoresis using the same procedure described for the antimicrobial resistance determinants.

### 2.10. Multilocus Sequence Typing (MLST)

KpSC isolates were genotyped by MLST analysis. Seven conserved housekeeping genes (*gap*A, *inf*B, *mdh*, *pgi*, *pho*E, *rpo*B, and *ton*B) were analyzed according to the MLST scheme described by Diancourt et al. [35]. Briefly, the target genes were amplified using PCR Master Mix (2x) (Thermo Fisher Scientific Baltics UAB, Vilnius, Lithuania) and the primers listed in Table S2. Amplifications were carried out with an initial denaturation at 95 °C for 3 min followed by 30 cycles of denaturation at 94 °C for 25 s, annealing at the specific temperature (Table S2) for 25 s, and extension at 72 °C for 1 min, with a final extension step at 72 °C for 5 min [19]. Amplicons were examined by agarose gel electrophoresis, purified, and subjected to cycle sequencing and Sanger sequencing as described above. The obtained sequences were analyzed to determine allele numbers for each housekeeping gene. The combination of alleles at the seven loci was used to assign a sequence type (ST) to each isolate. Allele numbers and STs were identified using the BIGSdb-Pasteur MLST database for the *Klebsiella pneumoniae* species complex [36].

### 2.11. Phylogenetic Analyses

Phylogenetic analyses were performed to explore sequence relationships among the KpSC isolates and selected reference sequences following 16S rRNA gene sequencing and MLST analysis. Sequence alignments were generated using the CLUSTAL_W algorithm implemented in the MEGA X software (version 10.0.4) [37]. Phylogenetic trees were constructed using the maximum likelihood method based on the Tamura–Nei substitution model [38]. The robustness of the inferred phylogenies was assessed by bootstrap analysis with 500 replicates.

Initially, a phylogenetic tree was generated based on the 16S rRNA gene sequences (~1500 bp). To improve species-level interpretation of the phylogeny, four reference sequences were retrieved from the GenBank database. These reference sequences represented *K. pneumoniae* (OR123585), *K. quasipneumoniae* (HG933296), and *K. variicola* (MK855126 and HG933294). A total of 10 sequences were included in the 16S rRNA gene-based phylogenetic analysis.

Another phylogenetic analysis was conducted based on the MLST data. For each isolate, a concatenated sequence (3012 bp) was generated by combining the allele sequences of the seven housekeeping genes. For comparative context, eight reference sequence types (STs) were obtained from the BIGSdb-Pasteur MLST database [36]. These included *K. pneumoniae* ST16, ST34, ST134, and ST1103; *K. quasipneumoniae* ST3212 and ST3215; and *K. variicola* ST197 and ST207. A total of 14 ST profiles were included in the MLST-based phylogenetic analysis.

### 2.12. Statistical Analysis

Phenotypic and genotypic findings for sequence-confirmed KpSC isolates were summarized descriptively as n/N (%). Given the limited and unequal representation of KpSC species, no inferential comparisons among species or phenotype–genotype association analyses were performed.

## 3. Results

### 3.1. Isolation and Identification of the Klebsiella pneumoniae Species Complex (KpSC)

A total of 68 presumptive mucoid Enterobacterales isolates were recovered from chicken (n = 61) and turkey (n = 7) fecal samples. Among an initial group of 10 presumptive mucoid isolates subjected directly to 16S rRNA and *rpoB* gene sequencing, isolates 16, 42, and 58 were confirmed as *K. pneumoniae*, isolate 43 as *K. quasipneumoniae*, and isolate 60 as *K. variicola*. Isolate 44 represented the same isolate as isolate 43 and was not counted separately. The other isolates in this initial group were not confirmed as KpSC members.

The remaining presumptive isolates were subsequently screened by MALDI-TOF MS, which identified isolates 34, 41, 51, 53, and 68 as potential KpSC members. Isolate 41 represented the same isolate as the previously sequence confirmed isolate 42 and was therefore not reanalyzed or counted separately. Subsequent 16S rRNA and *rpoB* gene sequencing did not confirm isolates 34, 51, and 53 as KpSC members, and these isolates were excluded. Isolate 68 was confirmed as *K. pneumoniae* and included in the study. Of the 419 fecal samples examined, the final dataset comprised six unique, sequence-confirmed KpSC isolates: *K. pneumoniae* (n = 4; isolates 16, 42, 58, and 68), *K. quasipneumoniae* (n = 1; isolate 43), and *K. variicola* (n = 1; isolate 60). The overall sample-level KpSC recovery proportion was 6/419 (1.43%). The progression from presumptive isolation to the final set of six unique sequence-confirmed KpSC isolates is summarized in Supplementary Table S4. Five of the six confirmed KpSC isolates originated from chicken samples, whereas one originated from a turkey sample.

Molecular analysis resolved all confirmed KpSC isolates at the species level, demonstrating complete concordance between the *rpo*B and 16S rRNA gene sequences. BLASTn analysis of both gene sequences revealed high similarity to reference sequences available in the GenBank database, ranging from 99.5% to 100.0%. The 16S rRNA gene sequences obtained in this study were deposited in the GenBank database and assigned ANs (Table 1). Following species confirmation by BLASTn analysis, the *rpo*B gene sequences were subsequently used for MLST analysis. Overall, six unique sequence-confirmed KpSC isolates representing three species were recovered, with concordant species assignments by 16S rRNA and *rpo*B sequence analyses.

### 3.2. Antimicrobial Susceptibility Testing

All six KpSC isolates were resistant to ampicillin, amoxicillin, and enrofloxacin (6/6, 100.0%). Resistance to trimethoprim/sulfamethoxazole was detected in two isolates (2/6, 33.3%), whereas resistance to gentamicin, nalidixic acid, doxycycline, and tetracycline was detected in one isolate for each antimicrobial agent (1/6, 16.7%). No resistance to kanamycin, ciprofloxacin, florfenicol, or chloramphenicol was detected (0/6, 0%).

At the isolate level, the single *K. quasipneumoniae* isolate (isolate 43) and the single *K. variicola* isolate (isolate 60) exhibited the same phenotype, with resistance to ampicillin, amoxicillin, and enrofloxacin and susceptibility to the remaining nine antimicrobial agents. Because each of these species was represented by a single isolate, these findings were not interpreted as species-level resistance patterns. Detailed isolate-level profiles are presented in Table 2 and Supplementary Table S3. Overall, all six isolates shared resistance to ampicillin, amoxicillin, and enrofloxacin, while resistance to the other tested antimicrobials was variable and confined to *K. pneumoniae* isolates.

### 3.3. Multidrug Resistance (MDR) and Multiple Antibiotic Resistance (MAR) Index

The MAR indices of the six KpSC isolates ranged from 0.25 to 0.67. Two of the six isolates (33.3%), both identified as *K. pneumoniae*, were classified as MDR based on resistance to antimicrobial agents from three or more classes. One of these isolates was resistant to three antimicrobial classes and had a MAR index of 0.33, whereas the other was resistant to five antimicrobial classes and had a MAR index of 0.67. The remaining four isolates, comprising two *K. pneumoniae*, one *K. quasipneumoniae*, and one *K. variicola* isolate, each had a MAR index of 0.25 (Figure 1). Overall, MDR was identified in two *K. pneumoniae* isolates, with MAR indices among the six isolates ranging from 0.25 to 0.67.

### 3.4. Phenotypic Analysis of Extended-Spectrum Beta Lactamase (ESBL) Activity

Phenotypic ESBL production was detected in one of the six isolates (1/6, 16.7%). This ESBL-positive isolate was *K. pneumoniae* isolate 68, which also exhibited the highest MAR index (0.67) and resistance to five antimicrobial classes. Phenotypic ESBL production was not detected by the method used in the remaining five isolates. Thus, phenotypic ESBL production was restricted to *K. pneumoniae* isolate 68.

### 3.5. Phenotypic Analysis of Carbapenemase Production

Phenotypic carbapenemase production was not detected by the method used in any of the six isolates (0/6, 0%). Thus, no phenotypic evidence of carbapenemase production was detected among the six KpSC isolates.

### 3.6. Genotypic Detection of Class 1 Integron (intI1), β-Lactamase Genes, PMQR, and Carbapenemase Genes

The most frequently detected determinants were *intI*1 and *bla*_SHV_, each identified in four of the six isolates (4/6, 66.7%). The *bla*_TEM_, *bla*_CTX-M_, and *qnr*B genes were each detected in two isolates (2/6, 33.3%), whereas *qnr*S was detected in one isolate (1/6, 16.7%). The remaining targeted resistance genes were not detected in any of the six isolates (0/6, 0%).

Among the four *K. pneumoniae* isolates, *intI*1 and *bla*_SHV_ were each detected in three isolates (3/4, 75.0%), while *bla*_TEM_ was detected in two isolates (2/4, 50.0%). The single *K. quasipneumoniae* isolate carried *intI*1 and *bla*_CTX-M_, whereas the single *K. variicola* isolate carried *bla*_SHV_ and *qnr*B. Detailed isolate-level profiles are presented in Table 3 and Supplementary Table S3. Overall, *intI*1 and *bla*_SHV_ were the most frequently detected determinants, while none of the targeted carbapenemase genes were detected.

### 3.7. Genotypic Detection of Virulence-Associated Genes

The *wabG* and *entB* genes were detected in all six isolates (6/6, 100.0%). The *kfu* gene was detected only in *K. pneumoniae* isolate 58 (1/6, 16.7%). The *rmp*A, *mag*A, *iuc*A, *iro*N, and *all*S genes were not detected in any of the six isolates (0/6, 0%). The distribution of the investigated virulence-associated genes is shown in Figure 2. Overall, *wab*G and *ent*B were detected in all six isolates, whereas *kfu* was detected in a single isolate, and the remaining targeted virulence-associated genes were not detected.

### 3.8. Multilocus Sequence Typing (MLST)

MLST identified six distinct sequence types among the six KpSC isolates. The four *K. pneumoniae* isolates were assigned to four different sequence types: ST15, ST292, ST5590, and ST5626. The *K. variicola* isolate was assigned to ST6479. In the chicken-origin *K. quasipneumoniae* isolate, a novel *rpo*B allele (allele 593) and the allelic profile 17-19-92-228-84-593-148 were identified. This profile was designated ST9812, and both the novel *rpo*B allele and ST9812 were registered in the Pasteur MLST database (Table 4). Furthermore, the MLST results corroborated the species identification obtained by 16S rRNA and *rpo*B gene sequence analyses. The species associated with the identified STs in the MLST database were fully consistent with the species assignments determined in this study.

Allelic variation was also examined across the seven housekeeping loci included in the MLST scheme. Among these genes, *inf*B exhibited the lowest level of polymorphism, with three alleles detected among the six isolates (50.0%). In contrast, *gap*A, *mdh*, and *rpo*B showed the highest degree of polymorphism, each displaying five different alleles (83.3%). The distribution of the observed alleles across the MLST loci is presented in Figure 3. Overall, MLST demonstrated sequence type diversity among the six isolates, with each isolate assigned to a distinct ST and the novel ST9812 identified in *K. quasipneumoniae*.

### 3.9. Phylogenetic Analyses

Phylogenetic analysis based on the 16S rRNA gene sequences revealed two major clusters (Figure 4). One cluster included the four *K. pneumoniae* isolates (PZ395315, PZ395316, PZ395318, and PZ395320) together with the *K. quasipneumoniae* isolate (PZ395317), whereas the *K. variicola* isolate (PZ395319) formed a separate cluster. The reference *K. variicola* sequences (MK855126 and HG933294) clustered with the study *K. variicola* isolate, while the reference *K. pneumoniae* (OR123585) and *K. quasipneumoniae* (HG933296) sequences grouped with the corresponding study isolates.

The phylogenetic tree based on the concatenated MLST sequences showed clustering consistent with species assignments (Figure 5). The *K. quasipneumoniae* isolate ST9812 clustered with the reference *K. quasipneumoniae* ST3212 and ST3215. The *K. variicola* isolate ST6479 clustered with the reference *K. variicola* ST197 and ST207, whereas the four *K. pneumoniae* isolates (ST15, ST292, ST5590, and ST5626) clustered with the reference *K. pneumoniae* ST16, ST34, ST134, and ST1103. Overall, the phylogenetic analyses supported the species assignments, with the MLST-based analysis showing clustering consistent with the identified KpSC species.

## 4. Discussion

The *Klebsiella pneumoniae* species complex (KpSC) is an ecologically flexible group capable of colonizing environmental habitats and the mucosal surfaces of humans and animals, including poultry [4,5,6]. Among its members, *K. pneumoniae* is an important opportunistic pathogen because of its antimicrobial resistance capacity and clinical relevance [1,2,3]. Previous studies have documented KpSC carriage and associated resistance genes in chicken and turkey populations [11,13,39]. The genetic plasticity of *K. pneumoniae* facilitates the acquisition and dissemination of MDR- and virulence-associated genes through mobile genetic elements and plasmids [40]. Therefore, characterization of antimicrobial susceptibility, virulence-associated genes, MLST profiles, and phylogenetic relationships can contribute to the evaluation of poultry-origin KpSC isolates within a One Health framework [9,11,19].

In the existing literature, studies on antimicrobial resistance- and virulence-associated with gene profiles in poultry have focused predominantly on *K. pneumoniae* because of its epidemiological and clinical importance. Conventional phenotypic methods and 16S rRNA sequencing may have limitations in distinguishing closely related KpSC members such as *K. variicola* and *K. quasipneumoniae*, which may therefore have been reported as *K. pneumoniae*. Although molecular approaches such as WGS and MLST can improve differentiation among KpSC members, *K. pneumoniae* remains the focus of current research. Consequently, the contribution of other KpSC members to the resistance pool in animal ecosystems remains less well characterized. Therefore, the findings for the KpSC isolates in this study are discussed in the context of the available *K. pneumoniae* literature.

In the present study, 68 isolates with presumptive mucoid Enterobacterales morphology were initially recovered, whereas only six unique isolates were confirmed as KpSC by sequence analysis, corresponding to a sample-level recovery proportion of 1.43% (6/419). MALDI-TOF MS screening showed that many of the presumptive non-KpSC isolates were identified as other Enterobacterales, predominantly *Enterobacter* spp., while *Escherichia coli* and a small number of other enteric bacteria, including *Citrobacter* sp., were also identified. These findings suggest that colony morphology on MacConkey and EMB agar alone may have limited specificity for presumptive KpSC identification. Subsequent molecular confirmation therefore improved identification specificity by excluding morphologically similar non-KpSC isolates.

Resistance to ampicillin and amoxicillin was observed in all six KpSC isolates (6/6, 100.0%). High ampicillin resistance has similarly been reported in poultry-origin isolates from Indonesia (97.5%), India (100%), and Egypt (100%) [41,42,43]. This finding is consistent with the intrinsic aminopenicillin resistance characteristics of KpSC.

All six KpSC isolates were resistant to enrofloxacin, whereas lower enrofloxacin resistance proportions have been reported in poultry-origin isolates from Indonesia (82.5%), China (63.64%), and Poland (16.37%) [13,19,42]. The factors underlying this finding could not be determined because reliable antimicrobial use records were unavailable. In contrast, all six isolates were susceptible to ciprofloxacin. The contrasting susceptibility results observed for enrofloxacin and ciprofloxacin should be interpreted cautiously because the underlying resistance mechanisms were not investigated comprehensively in the present study.

All six KpSC isolates were susceptible to kanamycin, florfenicol, and chloramphenicol. In contrast, poultry studies have reported 100% resistance to kanamycin and florfenicol in China [18], 81.82% resistance to chloramphenicol in China [15], 9.66% resistance to florfenicol in Poland [13], 100% resistance to florfenicol and chloramphenicol in diseased poultry in China [44], and 46.67% resistance to chloramphenicol in diseased poultry in Egypt [34]. Trimethoprim/sulfamethoxazole resistance was detected in two isolates (2/6, 33.3%), compared with 14.3% in Nigeria [45], 21.30% in Poland [13], and 72.73–100% in China [19,44]. These differences may reflect variation in geographical setting, poultry health status, study population, sample size, farm management, previous antimicrobial exposure, interpretive criteria, and laboratory methods.

The MAR indices of the six KpSC isolates ranged from 0.25 to 0.67, and two *K. pneumoniae* isolates were classified as MDR. The detection of MDR among poultry-origin KpSC isolates is relevant to antimicrobial resistance surveillance within a One Health context [11,19]. Higher MDR proportions have been reported in poultry-origin isolates from Indonesia (100%) [46] and China (87.88%, 48.39%, and 100%) [15,19,44]. However, direct comparisons are limited by differences in sample sizes, study populations, and antimicrobial susceptibility testing methods.

Phenotypic ESBL production was detected in one of the six KpSC isolates (1/6, 16.7%). ESBL positivity of 5% in healthy broiler cloacal swabs [47] and 9.8% in layer feces [48] has been reported, whereas higher proportions were reported in broiler feces in India (65.9%) [41] and broiler carcasses at slaughterhouses (96.7%) [17]. Differences among studies may reflect geographical setting, poultry health status, sample type, husbandry practices, previous antimicrobial exposure, and antimicrobial susceptibility testing methods; however, the contribution of these factors could not be determined from the available data.

Among the β-lactamase genes investigated, *bla*_SHV_ was the most frequently detected, being present in four of six isolates (66.7%), whereas *bla*_TEM_ and *bla*_CTX-M_ were each detected in two isolates (33.3%). High *bla*_SHV_ detection has also been reported in layer chicken feces (97.8%) [48], broiler farms (93.94%) [15], broiler carcasses (88.9%) [17], and internal organs of healthy poultry (100%) [49]. In contrast, other studies have reported *bla*_TEM_ or *bla*_CTX-M_ as the more frequently detected β-lactamase genes [16,42,50], indicating variation in β-lactamase gene profiles among poultry-origin isolates.

The phenotypically ESBL-positive *K. pneumoniae* isolate 68 carried *intI*1, *bla*_TEM_, *bla*_CTX-M_, and *bla*_SHV_ and had the highest MAR index (0.67), with resistance to five antimicrobial classes. Similar co-carriage of *bla*_SHV_, *bla*_CTX-M_, and *bla*_TEM_ has been reported in an ESBL-producing *K. pneumoniae* isolate from healthy broilers [47] and in broiler carcass isolates [17]. Class 1 integrons have also been detected in resistant animal-origin *Enterobacteriaceae* [50]. In the present study, *intI*1 and *bla*_CTX-M_ were additionally detected in the single *K. quasipneumoniae* isolate, whereas *bla*_SHV_ was detected in the single *K. variicola* isolate, documenting the presence of these resistance determinants in different KpSC members.

Phenotypic carbapenemase production was not detected by mCIM in any of the six KpSC isolates, and none of the targeted carbapenemase genes (*bla*_IMP-1_, *bla*_KPC-gp_, *bla*_NDM-1_, *bla*_OXA-48_, and *bla*_VIM_) were detected. Similar absence of carbapenem resistance has been reported in healthy poultry isolates from Norway [9,39] and healthy turkeys from Tunisia [51]. In contrast, carbapenem resistance or carbapenemase genes have been reported in poultry isolates from Egypt [12] and China [15,18], while metallo-β-lactamase-producing *K. pneumoniae* has been reported from poultry sources in Nigeria [52]. In Türkiye, carbapenem use in veterinary medicine is prohibited [53]; however, this regulatory context alone cannot explain the present findings. Interpretation is limited by the use of mCIM as the sole phenotypic carbapenemase assay and PCR screening restricted to five carbapenemase gene groups. Therefore, carbapenemase genes or variants outside the PCR panel and other carbapenem resistance mechanisms cannot be excluded.

Among the PMQR determinants investigated, *qnr*B was detected in two of the six isolates (33.3%), whereas *qnr*S was detected in one isolate (16.7%). Previous poultry studies have also reported higher detection of *qnr*B than *qnr*S, including Wu et al. [17], Li et al. [15], and Bhardwaj et al. [47], although the reported frequencies differed among studies. Saha et al. [16] also reported *qnr*B in poultry isolates. More recently, Kürekci et al. [26] detected *qnr*S and *qnr*B variants among tigecycline-resistant *K. pneumoniae* isolates recovered from poultry and chicken meat in Türkiye. The absence of *qnr*A in the present isolates is consistent with Bhardwaj et al. [47]; *qnr*A was also detected less frequently than *qnr*B in the studies of Wu et al. [17] and Li et al. [15]. Among the four *K. pneumoniae* isolates, *qnr*B and *qnr*S were detected in two isolates (2/4, 50.0%), while *qnr*B was also detected in the single *K. variicola* isolate, documenting the presence of this PMQR determinant in a poultry-origin *K. variicola* isolate.

Among the virulence-associated genes investigated, *wab*G and *ent*B were detected in all six KpSC isolates (6/6, 100.0%). The *wab*G gene is involved in lipopolysaccharide biosynthesis, whereas *ent*B is associated with enterobactin-mediated iron acquisition [19,20]. Similar findings have been reported by Hou et al. [19], who detected *wab*G and *ent*B in all isolates from healthy laying hens, and Khan et al. [14], who detected *ent*B in all healthy broiler isolates. In contrast, *kfu* was detected in only one *K. pneumoniae* isolate (1/6, 16.7%), compared with higher frequencies reported by Hou et al. [19] and Xue et al. [44]. Gene presence alone, however, does not demonstrate expression or functional activity.

None of the other investigated virulence-associated genes (*rmp*A, *mag*A, *iuc*A, *iro*N, *and all*S) were detected in the six isolates. In contrast, *rmp*A and *mag*A have been detected at higher frequencies in isolates from clinically diseased poultry in Egypt [34,43]. The absence of these major hypervirulence markers in the present isolates is consistent with findings reported in healthy livestock and poultry [14,19,47]. Thus, the examined isolates were characterized by the presence of *wab*G and *ent*B in all six isolates and the absence of the other investigated virulence-associated genes.

In this study, 16S rRNA sequencing successfully identified the confirmed KpSC isolates as *K. pneumoniae*, *K. quasipneumoniae*, and *K. variicola*, with species assignments fully concordant with those obtained by *rpo*B sequencing. Although limitations of 16S rRNA sequencing and 16S rRNA-based phylogenetic analysis in distinguishing closely related KpSC members have been reported [21], BLASTn analysis of the 16S rRNA sequences in the present study supported the species assignments obtained by *rpo*B sequencing. In the 16S-rRNA-based phylogenetic analysis, however, the *K. pneumoniae* and *K. quasipneumoniae* isolates grouped within the same major cluster, whereas the *K. variicola* isolate formed a separate cluster.

MLST is widely used to assess genetic diversity and clonal relationships among *Klebsiella* isolates [19,22,35]. In the present study, each of the six KpSC isolates was assigned to a distinct sequence type (ST15, ST292, ST5590, ST5626, ST6479, or ST9812), with the four *K. pneumoniae* isolates representing four different STs. Notably, a novel *rpo*B allele (allele 593) and the corresponding allelic profile 17-19-92-228-84-593-148, designated as ST9812, were identified in the single chicken-origin *K. quasipneumoniae* isolate and registered in the Pasteur MLST database. The identification and registration of ST9812 therefore represents a specific isolate-level contribution to the current knowledge of KpSC genetic diversity by adding a newly defined *K. quasipneumoniae* genotype to the publicly available MLST framework. This isolate carried *intI*1, *bla*_CTX-M_, *wab*G, and *ent*B but did not exhibit a phenotypic ESBL profile and lacked the targeted carbapenemase and major hypervirulence-associated genes. Although this finding expands the documented genetic diversity of poultry-associated KpSC and provides a traceable reference for future surveillance, its detection in a single isolate does not allow for conclusions regarding its distribution or epidemiological significance. Recovery and genomic characterization of additional ST9812 isolates from different sources will therefore be necessary to clarify its broader relevance.

Previous MLST studies have documented diverse sequence types among human clinical and animal-origin *K. pneumoniae* isolates, including ST14 [54] and ST11 [55] in human clinical settings and ST5387 and ST6085 in animal-origin isolates [19]. Of particular interest in the present study is the fact that the same ESBL-positive *K. pneumoniae* isolate 68, which was classified as MDR, had the highest MAR index (0.67), was resistant to five antimicrobial classes, was assigned to ST15, and carried *intI*1, *bla*_TEM_, *bla*_CTX-M_, *bla*_SHV_, *qnr*B, and *qnr*S. ST15 has been recognized as a globally disseminated high-risk clone associated with healthcare-associated infections and multidrug resistance [56] and has also been reported in companion animals, including dogs and cats [57]. Recent genomic evidence from Türkiye has also documented ST15 among avian-associated *K. pneumoniae*, with Kürekci et al. [26] identifying a single ST15 isolate in a WGS-based investigation of tigecycline-resistant *Klebsiella* spp. recovered from poultry and chicken meat. Its identification in a poultry-origin isolate represents an isolate-level surveillance observation but does not demonstrate clonal circulation between animal and human populations or establish poultry as a reservoir of this lineage. Comparative genomic and epidemiological data are required to investigate possible transmission relationships [25].

## 5. Conclusions

This study provides regional, isolate-level data on sequence-confirmed KpSC recovered from fecal samples of clinically healthy chickens and turkeys in Türkiye. The identification and registration of the novel *K. quasipneumoniae* ST9812, associated with the novel *rpo*B allele 593, represents a specific molecular contribution to the current knowledge of KpSC genetic diversity and provides a traceable reference for future surveillance. The six sequence-confirmed KpSC isolates recovered in this study showed distinct antimicrobial resistance, virulence-associated gene, and MLST profiles. MDR and ESBL-producing KpSC isolates carrying clinically relevant resistance determinants were identified among the recovered isolates; however, these findings do not establish broader circulation, poultry reservoir status, or zoonotic transmission. All six isolates carried *wab*G and *ent*B, whereas the other investigated virulence-associated genes were not detected. The identification of ST15 in a single *K. pneumoniae* isolate and the newly registered ST9812 in *K. quasipneumoniae* further illustrates the genetic heterogeneity of the recovered poultry-origin KpSC isolates. Further investigation using comparative whole-genome sequencing and larger isolate collections is needed to clarify the distribution and epidemiological significance of these findings.

## 6. Limitations

This study has several limitations. Convenience sampling, the single-province setting, uneven species representation, and the cross-sectional design may limit the broader generalizability of the findings. Reliable historical antimicrobial use data and quantitative information on biosecurity and stocking density were unavailable. In addition, the molecular analyses were restricted to the investigated resistance and virulence determinants and therefore do not exclude genes or mechanisms outside the tested targets.

## Figures and Tables

**Figure 1:**
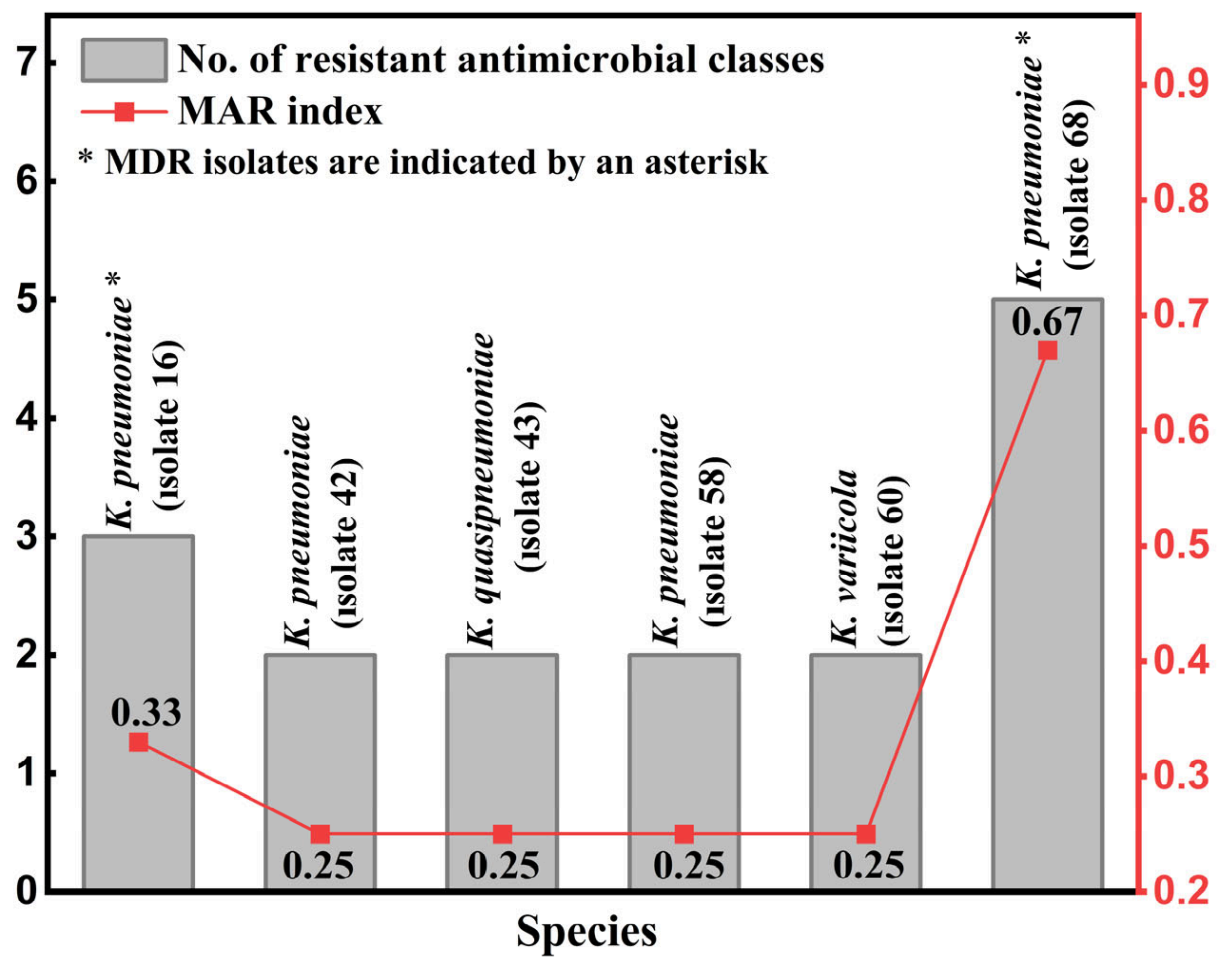
Antimicrobial resistance profiles and multiple antibiotic resistance (MAR) indices of the six KpSC isolates. Bars indicate the number of antimicrobial classes to which each isolate was resistant, and the line indicates the MAR index. The MAR index was calculated using the 12 individual antimicrobial agents tested as the denominator, whereas the bars represent the number of resistant antimicrobial classes. Asterisks indicate multidrug-resistant isolates.

**Figure 2:**
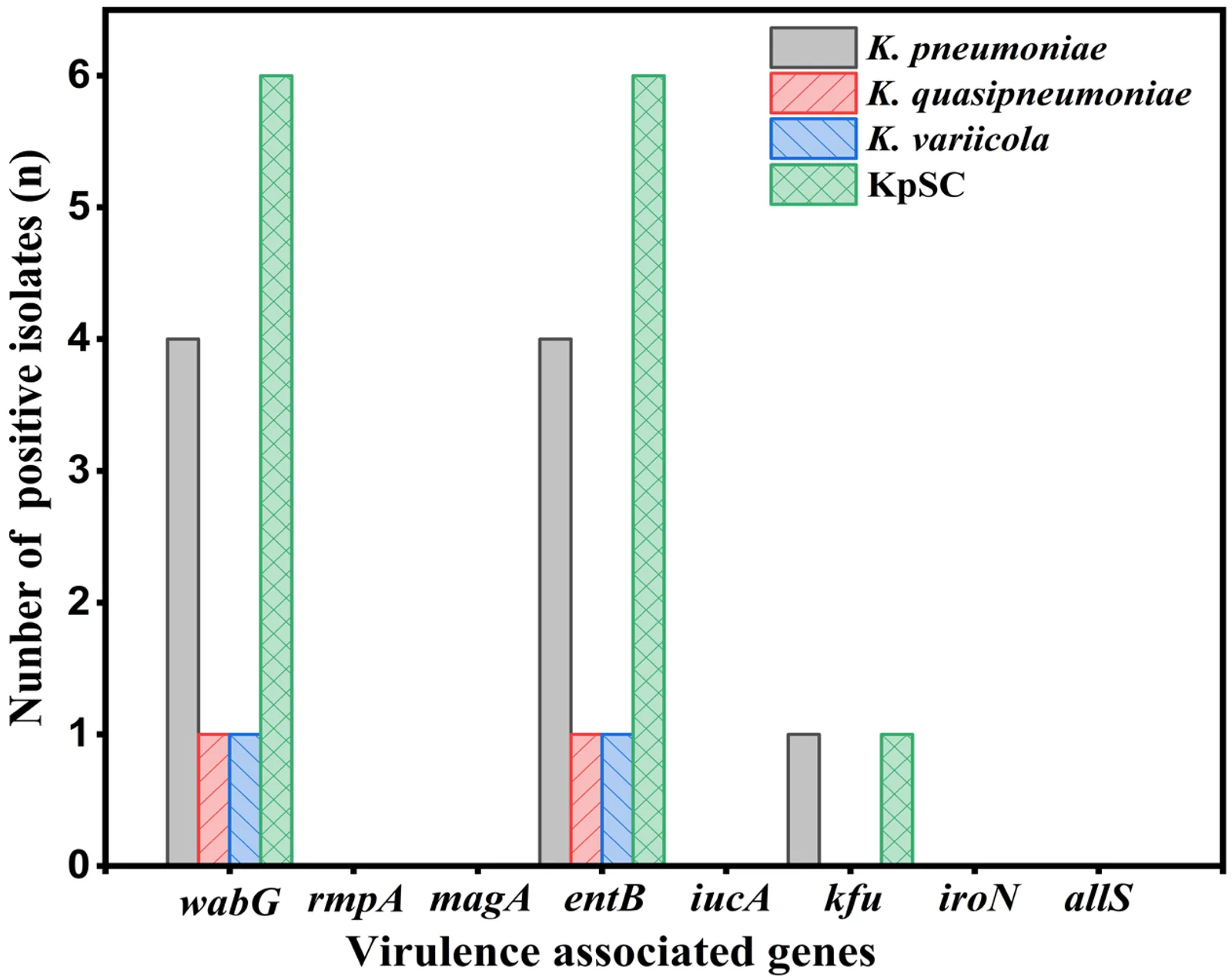
Number of KpSC isolates positive for the investigated virulence-associated genes (n = 6).

**Figure 3:**
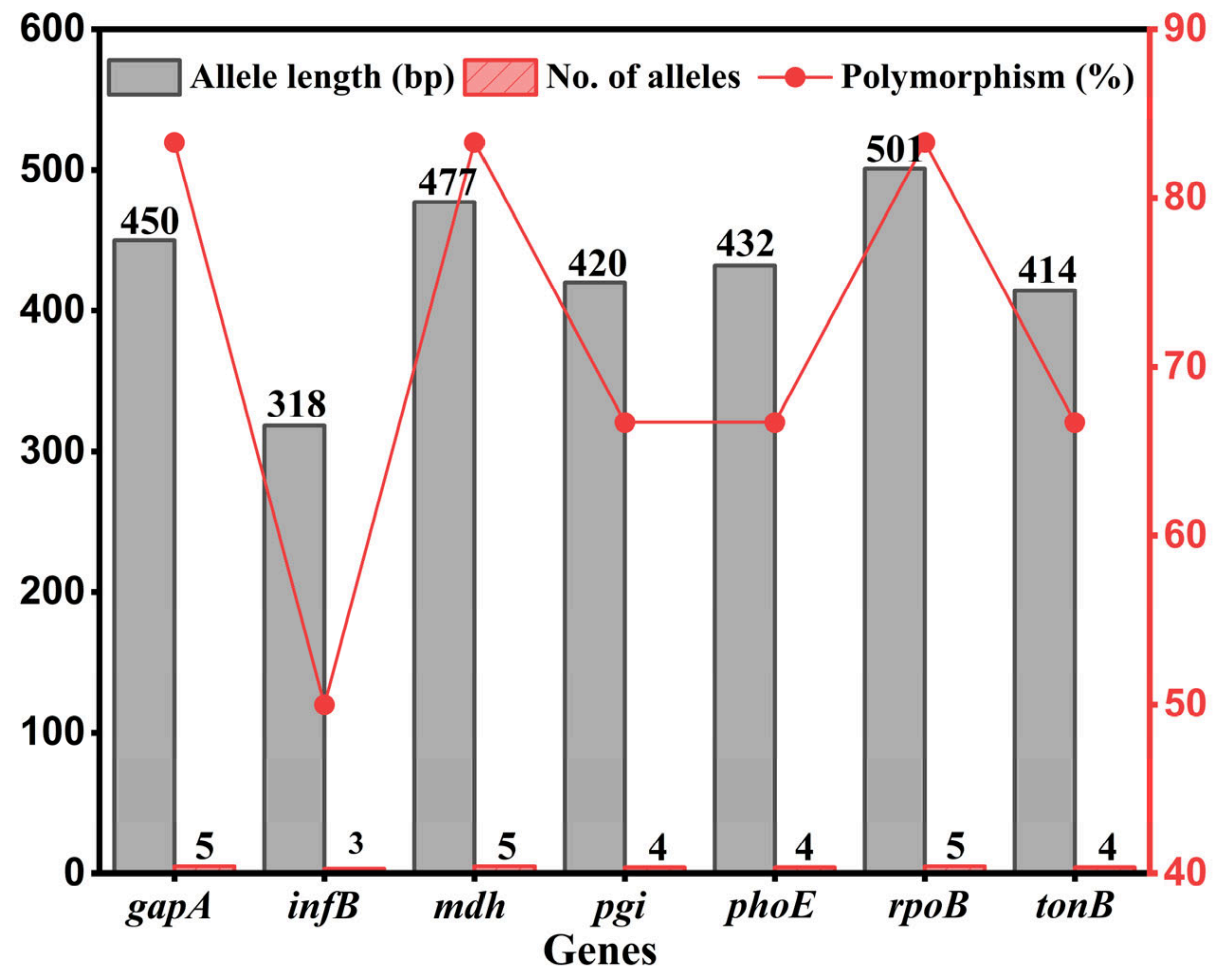
Allelic diversity of the seven housekeeping loci included in the KpSC MLST scheme.

**Figure 4:**
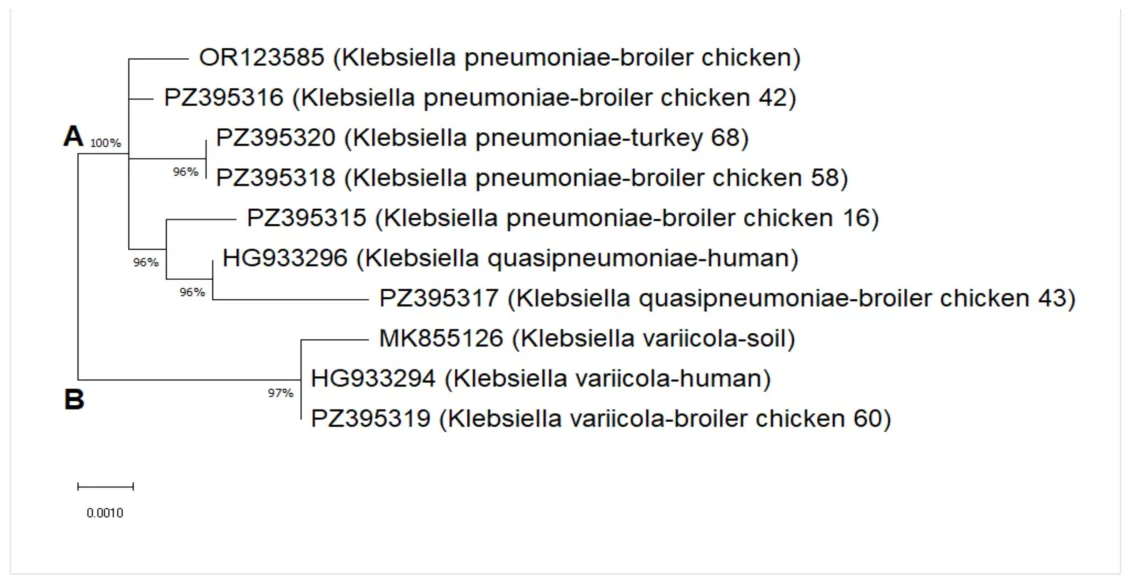
Maximum-likelihood phylogenetic tree based on 16S rRNA gene sequences of six KpSC isolates and four GenBank reference sequences. The tree was constructed using the Tamura–Nei model in MEGA X. Branch support was assessed with 500 bootstrap replicates.

**Figure 5:**
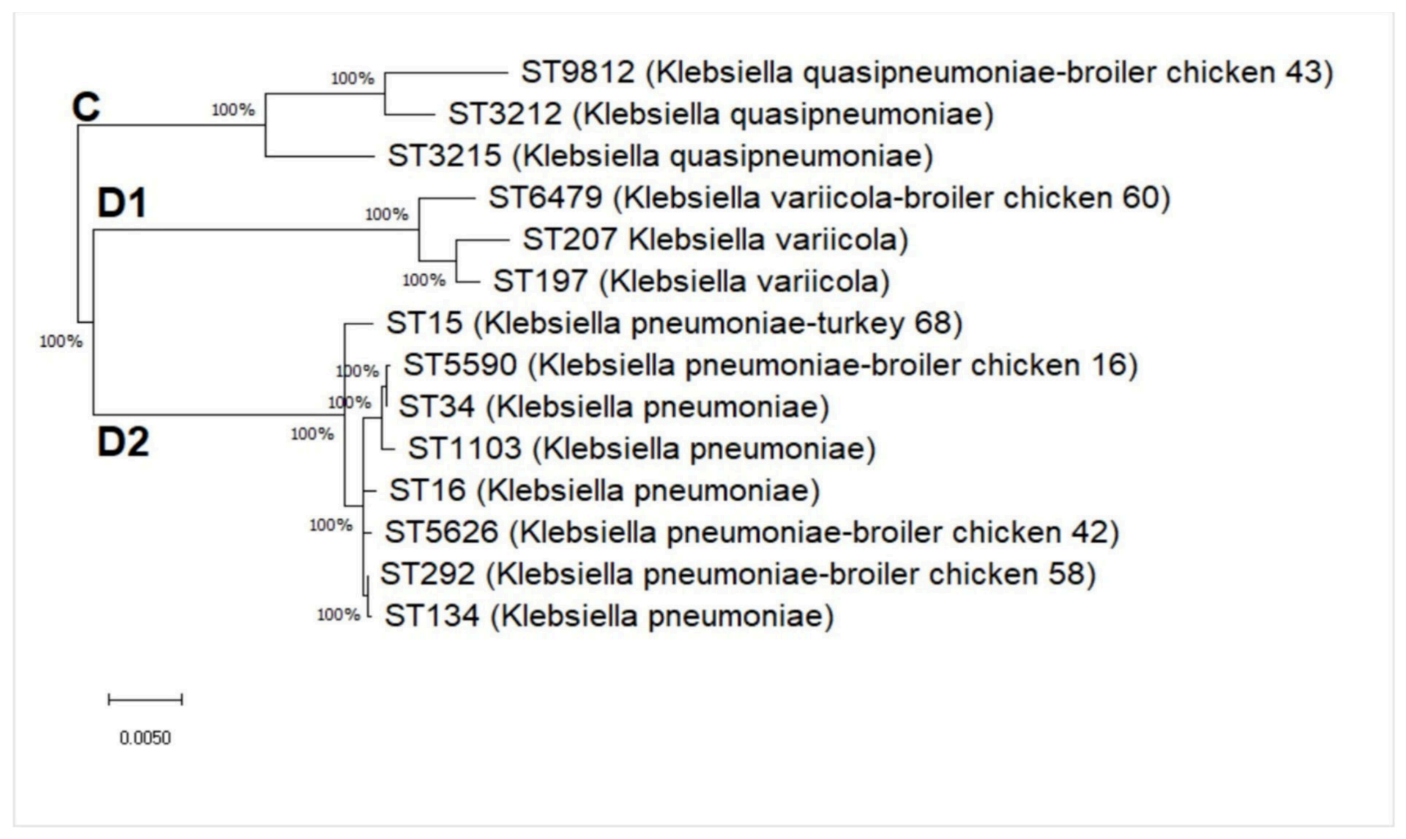
Maximum-likelihood phylogenetic tree based on concatenated MLST sequences of six KpSC isolates and eight reference STs. The alignment contained 3012 nucleotide positions. The tree was constructed using the Tamura–Nei model in MEGA X with 500 bootstrap replicates.

**Table 1:** Species identification of KpSC isolates by *rpo*B and 16S rRNA gene sequencing and corresponding GenBank accession numbers.

Isolate ID	*rpo*B Gene	16S rRNA Gene	GenBank Accession Number
16	*K. pneumoniae*	*K. pneumoniae*	PZ395315
42	*K. pneumoniae*	*K. pneumoniae*	PZ395316
43	*K. quasipneumoniae*	*K. quasipneumoniae*	PZ395317
58	*K. pneumoniae*	*K. pneumoniae*	PZ395318
60	*K. variicola*	*K. variicola*	PZ395319
68	*K. pneumoniae*	*K. pneumoniae*	PZ395320

**Table 2:** Phenotypic antimicrobial resistance profiles of poultry-origin KpSC isolates.

Antimicrobial Class	Antimicrobial Agent	*K. pneumoniae* (n = 4)	*K. quasipneumoniae* (n = 1)	*K. variicola* (n = 1)	Total, n/N (%) (n = 6)
Aminopenicillins	ampicillin	4/4 (100.0)	1/1 (100.0)	1/1 (100.0)	6/6 (100.0)
	amoxicillin	4/4 (100.0)	1/1 (100.0)	1/1 (100.0)	6/6 (100.0)
Aminoglycosides	gentamicin	1/4 (25.0)	0/1 (0)	0/1 (0)	1/6 (16.7)
	kanamycin	0/4 (0)	0/1 (0)	0/1 (0)	0/6 (0)
Quinolones and fluoroquinolones	enrofloxacin	4/4 (100.0)	1/1 (100.0)	1/1 (100.0)	6/6 (100.0)
	ciprofloxacin	0/4 (0)	0/1 (0)	0/1 (0)	0/6 (0)
	nalidixic acid	1/4 (25.0)	0/1 (0)	0/1 (0)	1/6 (16.7)
Phenicols	florfenicol	0/4 (0)	0/1 (0)	0/1 (0)	0/6 (0)
	chloramphenicol	0/4 (0)	0/1 (0)	0/1 (0)	0/6 (0)
Tetracyclines	doxycycline	1/4 (25.0)	0/1 (0)	0/1 (0)	1/6 (16.7)
	tetracycline	1/4 (25.0)	0/1 (0)	0/1 (0)	1/6 (16.7)
Folate pathway antagonists	trimethoprim/sulfamethoxazole	2/4 (50.0)	0/1 (0)	0/1 (0)	2/6 (33.3)

Values are presented as n/N (%). Because *K. quasipneumoniae* and *K. variicola* were each represented by a single isolate, the corresponding values are descriptive and should not be interpreted as evidence of species-level resistance patterns.

**Table 3:** Antimicrobial resistance determinants detected in poultry-origin KpSC isolates.

Determinant Group	Resistance Determinant	*K. pneumoniae* (n = 4)	*K. quasipneumoniae* (n = 1)	*K. variicola* (n = 1)	Total, n/N (%) (n = 6)
Class I Integron	*intI*1	3/4 (75.0)	1/1 (100.0)	0/1 (0)	4/6 (66.7)
β-Lactams	*bla*_TEM_	2/4 (50.0)	0/1 (0)	0/1 (0)	2/6 (33.3)
	*bla*_CTX-M_	1/4 (25.0)	1/1 (100.0)	0/1 (0)	2/6 (33.3)
	*bla*_SHV_	3/4 (75.0)	0/1 (0)	1/1 (100.0)	4/6 (66.7)
	*bla*_OXA-1_	0/4 (0)	0/1 (0)	0/1 (0)	0/6 (0)
Quinolones	*qnr*A	0/4 (0)	0/1 (0)	0/1 (0)	0/6 (0)
	*qnr*B	1/4 (25.0)	0/1 (0)	1/1 (100.0)	2/6 (33.3)
	*qnr*S	1/4 (25)	0/1 (0)	0/1 (0)	1/6 (16.7)
	*qep*A	0/4 (0)	0/1 (0)	0/1 (0)	0/6 (0)
Carbapenems	*bla*_KPC-gp_	0/4 (0)	0/1 (0)	0/1 (0)	0/6 (0)
	*bla*_NDM-1_	0/4 (0)	0/1 (0)	0/1 (0)	0/6 (0)
	*bla*_IMP-1_	0/4 (0)	0/1 (0)	0/1 (0)	0/6 (0)
	*bla*_VIM_	0/4 (0)	0/1 (0)	0/1 (0)	0/6 (0)
	*bla*_OXA-48_	0/4 (0)	0/1 (0)	0/1 (0)	0/6 (0)

Values are presented as n/N (%). Because *K. quasipneumoniae* and *K. variicola* were each represented by a single isolate, the corresponding values are descriptive and should not be interpreted as evidence of species-level differences.

**Table 4:** MLST profiles of poultry-origin KpSC isolates.

Isolate ID	Species	*gap*A	*inf*B	*mdh*	*pho*E	*pgi*	*rpo*B	*ton*B	ST
16	*K. pneumoniae*	2	3	435	9	1	7	4	5590
42	*K. pneumoniae*	4	1	2	1	3	4	4	5626
43	*K. quasipneumoniae*	17	19	92	228	84	**593**	148	**9812**
58	*K. pneumoniae*	2	1	2	1	1	1	4	292
60	*K. variicola*	16	1	21	49	39	22	67	6479
68	*K. pneumoniae*	1	1	1	1	1	1	1	15

Bold values indicate the novel *rpo*B allele (593) and the novel sequence type ST9812.

## Data Availability

The original contributions presented in the study are included in the article/Supplementary Materials. Further inquiries can be directed to the corresponding author. The 16S rRNA gene sequences generated in this study are openly available in GenBank under accession numbers PZ395315–PZ395320. The novel *rpoB* al-lele (allele 593) and the corresponding sequence type ST9812 are publicly available in the Institut Pasteur BIGSdb Klebsiella pneumoniae species complex MLST database (https://bigsdb.pasteur.fr/klebsiella/, accessed on 9 June 2026). Additional data are available from the corresponding author upon reasonable request.

## Supplementary Materials

The following supporting information can be downloaded at: https://www.mdpi.com/article/10.3390/microorganisms14102138/s1. Table S1: Number and poultry origin of the fecal samples collected for KpSC isolation. Table S2: Primer sequences, amplicon sizes, annealing temperatures, PCR cycling profiles, and primer references for the 16S rRNA gene, *intI*1, antimicrobial resistance determinants, virulence associated genes, and MLST genes. Table S3: Molecular characterization, antimicrobial resistance, and virulence associated gene profiles of poultry origin KpSC isolates. Table S4: Isolate tracking and identification workflow from presumptive mucoid Enterobacterales isolates to the final sequence confirmed KpSC dataset. References [15,19,28,58,59,60,61,62,63,64] are cited in the Supplementary Materials.

## Abbreviations

KpSC	*Klebsiella pneumoniae* species complex
AMR	Antimicrobial resistance
MDR	Multidrug resistance
MAR Index	Multiple antibiotic resistance
ESBL	Extended-spectrum beta lactamase
PMQR	Plasmid-mediated quinolone resistance
MLST	Multilocus sequence typing
MALDI-TOF MS	Matrix-assisted laser desorption/ionization time-of-flight mass spectrometry

## References

[1] Ochońska D., Brzychczy-Włoch M. (2024). *Klebsiella pneumoniae*–taxonomy, occurrence, identification, virulence factors and pathogenicity. Adv. Microbiol..

[2] Mohammadpour D., Memar M.Y., Leylabadlo H.E., Ghotaslou A., Ghotaslou R. (2025). Carbapenem-resistant *Klebsiella pneumoniae*: A comprehensive review of phenotypic and genotypic methods for detection. Microbe.

[3] Shah A.A., Alwashmi A.S., Abalkhail A., Alkahtani A.M. (2025). Emerging challenges in *Klebsiella pneumoniae*: Antimicrobial resistance and novel approach. Microb. Pathog..

[4] Asokan S., Jacob T., Jacob J., AlSosowaa A.A., Cherian T., Peijnenburg W.J.G.M., Vijayan S. (2025). *Klebsiella pneumoniae*: A growing threat in the era of antimicrobial resistance. Microbe.

[5] Barbier E., Rodrigues C., Depret G., Passet V., Gal L., Piveteau P., Brisse S. (2020). The ZKIR assay, a real-time PCR method for the detection of *Klebsiella pneumoniae* and closely related species in environmental samples. Appl. Environ. Microbiol..

[6] Wyres K.L., Lam M.M.C., Holt K.E. (2020). Population genomics of *Klebsiella pneumoniae*. Nat. Rev. Microbiol..

[7] McAndrew G., Barbier E., Rodrigues C., Piveteau P., Brisse S., Reddington K. (2025). KpSC-ID: A multiplex real-time PCR assay for the simultaneous detection of the *Klebsiella pneumoniae* species complex and specific identification of *Klebsiella pneumoniae*, Klebsiella quasipneumoniae and Klebsiella variicola. Microbiology.

[8] Rodrigues C., Hauser K., Cahill N., Ligowska-Marzęta M., Centorotola G., Cornacchia A., Fierro R.G., Haenni M., Nielsen E.M., Piveteau P. (2022). High prevalence of *Klebsiella pneumoniae* species complex in European food products: A multicentric study comparing culture and molecular detection methods. Microbiol. Spectr..

[9] Kaspersen H., Urdahl A.M., Franklin-Alming F.V., Ilag H.K., Hetland M.A.K., Bernhoff E., Löhr I.H., Sunde M. (2023). Population dynamics and characteristics of *Klebsiella pneumoniae* from healthy poultry in Norway. Front. Microbiol..

[10] World Health Organization (2024). WHO Bacterial Priority Pathogens List, 2024: Bacterial Pathogens of Public Health Importance, to Guide Research, Development and Strategies to Prevent and Control Antimicrobial Resistance.

[11] Kot B., Witeska M. (2024). Review: Antimicrobial resistance of *Klebsiella pneumoniae* isolated from poultry, cattle and pigs. Animal.

[12] Hamza E., Dorgham S.M., Hamza D.A. (2016). Carbapenemase-producing *Klebsiella pneumoniae* in broiler poultry farming in Egypt. J. Glob. Antimicrob. Resist..

[13] Kowalczyk J., Czokajło I., Gańko M., Śmiałek M., Koncicki A. (2022). Identification and antimicrobial resistance in *Klebsiella* spp. isolates from turkeys in Poland between 2019 and 2022. Animals.

[14] Khan M.M., Mushtaq M.A., Abbas N., Fatima F., Gibbon M.J., Schierack P., Mohsin M. (2024). Occurrence, antimicrobial resistance and genomic features of *Klebsiella pneumoniae* from broiler chicken in Faisalabad, Pakistan. Front. Vet. Sci..

[15] Li Z., Xin L., Peng C., Liu C., Wang P., Yu L., Liu M., Wang F. (2022). Prevalence and antimicrobial susceptibility profiles of ESBL-producing *Klebsiella pneumoniae* from broiler chicken farms in Shandong Province, China. Poult. Sci..

[16] Saha O., Basri R., Hossain M.A., Sultana M. (2023). Characterization of multidrug and heavy metal resistance of carbapenemase-producing *Klebsiella pneumoniae* from poultry samples in Bangladesh. Vet. Med. Sci..

[17] Wu H., Wang M., Liu Y., Wang X., Wang Y., Lu J., Xu H. (2016). Characterization of antimicrobial resistance in *Klebsiella* species isolated from chicken broilers. Int. J. Food Microbiol..

[18] Xiao X., Yang P., Peng K., Li Y., Wang Q., Lv Y., Feil E., Wang Z., Li R. (2025). Genomic diversity and coexistence of multidrug-resistance mechanisms of *Klebsiella pneumoniae* in poultry farms. Transbound. Emerg. Dis..

[19] Hou G., Ahmad S., Li Y., Yan D., Yang S., Chen S., Qiu Z., Yu X., Li N., Li Y. (2024). Epidemiological, virulence, and antibiotic resistance analysis of *Klebsiella pneumoniae*, a major source of threat to livestock and poultry in some regions of Xinjiang, China. Animals.

[20] Monteiro A.D.S.S., Cordeiro S.M., Reis J.N. (2024). Virulence Factors in *Klebsiella pneumoniae*: A Literature Review. Indian J. Microbiol..

[21] Galib M., Nguyen V.H. (2025). Identification of robust species-specific marker genes for the demarcation of the *Klebsiella pneumoniae*–*quasipneumoniae–variicola* complex. J. Med. Microbiol..

[22] Zhang L.-Y., Tian B., Huang Y.-H., Gu B., Ju P., Luo Y., Tang J., Wang L. (2023). Classification and prediction of *Klebsiella pneumoniae* strains with different MLST allelic profiles via SERS spectral analysis. PeerJ.

[23] Kaya S., Sesli Çetin E., Arıkan S., Tetik T., Kesbiç H., Yaşar S. (2007). Tavuklardan izole edilen *E. coli*, *Klebsiella* ve enterokoklarda antibiotik duyarlılık durumları. Med. J. Süleyman Demirel Univ..

[24] Uyanik T., Gülel G.T., Alışarlı M. (2021). Characterization of extended-spectrum beta-lactamase-producing Enterobacterales from organic and conventional chicken meats. Lett. Appl. Microbiol..

[25] Kürekci C., Ünaldı Ö., Şahin S., García-Meniño I., Hammerl J.A. (2024). Impact and diversity of ESBL-producing *Klebsiella pneumoniae* recovered from raw chicken meat samples in Türkiye. Antibiotics.

[26] Kürekci C., Lu X., Şahin S., Soyer Y., Wang Z., Hammerl J.A., Li R. (2026). Whole-Genome Insights Into Tigecycline-Resistant *Klebsiella* Spp. Harboring Colistin Resistance Gene From Food Animals in Türkiye. Transbound. Emerg. Dis..

[27] Markey B., Leonard F., Archambault M., Cullinane A., Maguire D. (2013). Enterobacteriaceae. Clinical Veterinary Microbiology.

[28] Lane D.J., Stackebrandt E., Goodfellow M. (1991). 16S/23S rRNA sequencing. Nucleic Acid Techniques in Bacterial Systematics.

[29] Sambrook J., Russell D.W. (2002). Molecular Cloning: A Laboratory Manual.

[30] Clinical and Laboratory Standards Institute (2023). Performance Standards for Antimicrobial Susceptibility Testing.

[31] Magiorakos A.P., Srinivasan A., Carey R.B., Carmeli Y., Falagas M.E., Giske C.G., Harbarth S., Hindler J.F., Kahlmeter G., Olsson-Liljequist B. (2012). Multidrug-resistant, extensively drug-resistant and pandrug-resistant bacteria: An international expert proposal for interim standard definitions for acquired resistance. Clin. Microbiol. Infect..

[32] Xiang Y., Wei Z., Mo X., Liao Y., Wen C., Liao M., Wei J., Lin C., Li W., Chen J. (2026). Multidimensional analysis of pathogen epidemic patterns, their correlation with milk quality, and drug resistance and virulence traits of dominant pathogenic *Escherichia coli* in high-somatic-cell buffalo milk: Guangxi, China. Food Microbiol..

[33] Krumperman P.H. (1983). Multiple antibiotic resistance indexing of *Escherichia coli* to identify high-risk sources of fecal contamination of foods. Appl. Environ. Microbiol..

[34] Younis G., Awad A., El-Gamal A., Hosni R. (2016). Virulence properties and antimicrobial susceptibility profiles of *Klebsiella* species recovered from clinically diseased broiler chicken. Adv. Anim. Vet. Sci..

[35] Diancourt L., Passet V., Verhoef J., Grimont P.A.D., Brisse S. (2005). Multilocus sequence typing of *Klebsiella pneumoniae* nosocomial isolates. J. Clin. Microbiol..

[36] Institut Pasteur BIGSdb-Pasteur: *Klebsiella pneumoniae*. Species Complex.

[37] Kumar S., Stecher G., Li M., Knyaz C., Tamura K. (2018). MEGA X: Molecular evolutionary genetics analysis across computing platforms. Mol. Biol. Evol..

[38] Tamura K., Nei M. (1993). Estimation of the number of nucleotide substitutions in the control region of mitochondrial DNA in humans and chimpanzees. Mol. Biol. Evol..

[39] Franklin-Alming F.V., Kaspersen H., Hetland M.A.K., Bakksjø R.-J., Nesse L.L., Leangapichart T., Löhr I.H., Telke A.A., Sunde M. (2021). Exploring *Klebsiella pneumoniae* in healthy poultry reveals high genetic diversity, good biofilm-forming abilities and higher prevalence in turkeys than broilers. Front. Microbiol..

[40] Han B., Feng C., Jiang Y., Ye C., Wei Y., Liu J., Zeng Z. (2025). Mobile genetic elements encoding antibiotic resistance genes and virulence genes in *Klebsiella pneumoniae*: Important pathways for the acquisition of virulence and resistance. Front. Microbiol..

[41] Parmar N., Singh R., Kaur S., Tyagi A., Malik H., Aulakh R.S., Gill J.P.S. (2025). Unraveling genetic similarities of *Klebsiella pneumoniae* from poultry and poultry handlers in Punjab, India. Gene Rep..

[42] Safika S., Nilasari Z., Pasaribu F.H. (2022). Detection of antibiotic resistance coding gene in *Klebsiella pneumoniae* bacteria isolated from broiler chickens in West Java, Indonesia. J. Appl. Pharm. Sci..

[43] Enany M.E., El-Mahallawy H.S., Mohammed W.E., Hassan W.M., Aboueisha A.M. (2025). Emergence of extensively drug-resistant *Klebsiella pneumoniae* from broiler chickens and clinical samples in Egypt. BMC Microbiol..

[44] Xue Y., Shi F., Zhou B., Shi Y., Luo W., Zhu J., Yang Y., Chen S., Qin T., Peng D. (2025). Biofilm formation, antibiotic resistance, and virulence analysis of human and avian origin *Klebsiella pneumoniae* from Jiangsu, China. Vet. Sci..

[45] Bamidele O., Yakubu A., Joseph E.B., Amole T.A. (2022). Antibiotic resistance of bacterial isolates from smallholder poultry droppings in the Guinea savanna zone of Nigeria. Antibiotics.

[46] Hayati M., Indrawati A., Mayasari N.L.P.I., Istiyaningsih I., Atikah N. (2019). Molecular detection of extended-spectrum β-lactamase-producing *Klebsiella pneumoniae* isolates of chicken origin from East Java, Indonesia. Vet. World.

[47] Bhardwaj K., Shenoy M.S., Baliga S., Unnikrishnan B., Baliga B.S., Shetty V.K. (2021). Characterization of antibiotic-resistant phenotypes and linked genes of *Escherichia coli* and *Klebsiella pneumoniae* from healthy broiler chickens, Karnataka, India. Poult. Sci..

[48] Tian R., Yang M., Yang Y., Xu S., Chen W., Xia L. (2026). Emergence and genomic characterization of mcr-8.2-positive *Klebsiella pneumoniae* from layer chickens in Xinjiang, China. Poult. Sci..

[49] Abd El-Tawab A., Soliman E., El-Dahshan E.S., El-Bery A. (2022). Molecular studies on some antibiotic-resistant genes of *Klebsiella* species isolated from chicken. Benha Vet. Med. J..

[50] Ejaz H., Younas S., Abosalif K.O.A., Junaid K., Alzahrani B., Alsrhani A., Abdalla A.E., Ullah M.I., Qamar M.U., Hamam S.S.M. (2021). Molecular analysis of *bla*_SHV_, *bla*_TEM_, and *bla*_CTX-M_ in extended-spectrum β-lactamase producing *Enterobacteriaceae* recovered from fecal specimens of animals. PLoS ONE.

[51] Romdhani A., Dhaouadi S., Cheriet S., Lengliz S., Boutiba-Ben Boubaker I., Landolsi R.B., Abbassi M.S. (2025). Genetic characterization of extended-spectrum beta-lactamase-producing *Escherichia coli* and *Klebsiella pneumoniae* collected from healthy turkeys. J. Infect. Dev. Ctries..

[52] Ejikeugwu C., Nworie O., Saki M., Al-Dahmoshi H.O.M., Al-Khafaji N.S.K., Ezeador C., Nwakaeze E., Eze P., Oni E., Obi C. (2021). Metallo-β-lactamase and *Amp*C genes in *Escherichia coli*, *Klebsiella pneumoniae*, and *Pseudomonas aeruginosa* isolates from abattoir and poultry origin in Nigeria. BMC Microbiol..

[53] Republic of Türkiye Ministry of Agriculture and Forestry İnsanlarda Belirli Enfeksiyonların Tedavisi İçin Ayrılmış Antimikrobiyallerin Veya Antimikrobiyal Gruplarının Belirlenmesi Hakkında Tebliğ (Tebliğ No: 2024/33). Resmî Gazete, 18 December 2024, No. 32756. https://www.resmigazete.gov.tr/eskiler/2024/12/20241218-2.htm.

[54] Al-Zahrani I.A., Aljabri A., Alhazmi W.A., Yasir M., Abujamel T., Alghamdi A.K., Azhar E.I. (2024). Genomic analysis of extensively drug resistant (XDR) *Klebsiella pneumoniae* high-risk clone ST14 co-harboring *bla*_NDM_ and *bla*_OXA-48_ recovered from Saudi Arabia. J. Infect. Public Health.

[55] Shi Q., Ruan Z., Zhang P., Hu H., Han X., Wang Z., Lou T., Quan J., Lan W., Weng R. (2024). Epidemiology of carbapenem-resistant *Klebsiella pneumoniae* in China and the evolving trends of predominant clone ST11: A multicentre, genome-based study. J. Antimicrob. Chemother..

[56] Tajuelo A., Gato E., Moya C., Prieto Martín-Gil S., Cano-Castaño B., Vaughan L., Miguela-Villoldo P., Pérez-Vázquez M., Moscoso M., Rodiño-Janeiro B.K. (2026). KpFhaB/FhaC is a virulence-associated TPS system of the globally-disseminated *Klebsiella pneumoniae* ST15 high-risk clone. Front. Cell. Infect. Microbiol..

[57] Fordham S.M.E., Sheridan E., Drobniewski F. (2026). Companion Animals Harbour Globally Circulating Human-Associated *Klebsiella pneumoniae* Lineages and High-Risk Antimicrobial Resistance Clones. Transbound. Emerg. Dis..

[58] Guerra B., Junker E., Miko A., Helmuth R., Mendoza M.C. (2004). Characterization and localization of drug resistance determinants in multidrug-resistant, integron-carrying *Salmonella enterica* serotype Typhimurium strains. Microb. Drug Resist..

[59] Arlet G., Philippon A. (1991). Construction by polymerase chain reaction and intragenic DNA probes for three main types of transferable β-lactamases (TEM, SHV, CARB). FEMS Microbiol. Lett..

[60] Batchelor M., Hopkins K., Threlfall E.J., Clifton-Hadley F.A., Stallwood A.D., Davies R.H., Liebana E. (2005). *bl*a_CTX-M_ genes in clinical *Salmonella* isolates recovered from humans in England and Wales from 1992 to 2003. Antimicrob. Agents Chemother..

[61] Arlet G., Rouveau M., Philippon A. (1997). Substitution of alanine for aspartate at position 179 in the SHV-6 extended-spectrum β-lactamase. FEMS Microbiol. Lett..

[62] Colom K., Pérez J., Alonso R., Fernández-Aranguiz A., Lariño E., Cisterna R. (2003). Simple and reliable multiplex PCR assay for detection of *bla*_TEM_, *bla*_SHV_ and *bla*_OXA-1_ genes in *Enterobacteriaceae*. FEMS Microbiol. Lett..

[63] Robicsek A., Strahilevitz J., Sahm D.F., Jacoby G.A., Hooper D.C. (2006). *qnr* prevalence in ceftazidime-resistant *Enterobacteriaceae* isolates from the United States. Antimicrob. Agents Chemother..

[64] Yamane K., Wachino J.-I., Suzuki S., Kimura K., Shibata N., Kato H., Shibayama K., Konda T., Arakawa Y. (2007). New plasmid-mediated fluoroquinolone efflux pump, *Qep*A, found in an *Escherichia coli* clinical isolate. Antimicrob. Agents Chemother..

