# Supplementary material for: Phenotypic and Molecular Characterization of Klebsiella pneumoniae Species Complex Isolates from Clinically Healthy Poultry, with Identification of Novel K. quasipneumoniae ST9812
Source: Microorganisms. 2026 Sep 23;14(10):2138. doi: 10.3390/microorganisms14102138 (PMC13707530; doi:10.3390/microorganisms14102138)
Supplement: Supplementary file 1 [file microorganisms-14-02138-s001.zip › microorganisms-4561713-supplementary.pdf]

Table S1: Number and poultry origin of the fecal samples collected for KpSC isolation.

Poultry Origin	Province	District	Village	Number of farms	Number of materials	Total
Chicken	Diyarbakır	Çınar	village 1	9	100	351
			village 2	14	94	
		Bağlar	village 1	5	35	
		Central District	village 1	1	72	
			village 2	1	8	
			village 3	2	42	
turkey	Diyarbakır	Central District	village 1	1	19	68
			village 2	1	49	
			**Total**			**419**

Table S2: Primer sequences, amplicon sizes, annealing temperatures, PCR cycling profiles, and primer references for the 16S rRNA gene, *intI*1, antimicrobial resistance determinants, virulence-associated genes, and MLST genes.

Gene	Primer Sequence (5'-3')	Size (bp)	Annealing Temparature (◦C)	PCR Profile	Primer Reference
	16S rRNA gene				
**16S rRNA**	27F: AGAGTTTGATCCTGGCTCAG<br>1492R: TACGGYTACCTTGTTACGACTT	~1500	50	A	[28]
	Integrase 1 gene				
*intI*1	F-GCCTTGCTGTTCTTCTAC<br>R-GATGCCTGCTTGTTCTAC	558	54	B	[58]
	β-Lactamase Genes				
bla$_{TEM}$	F: TTGGGTGCACGAGTGGGTTA<br>R: TAATTGTTGCCGGGAAGCTA	506	54	B	[59]
bla$_{CTX-M}$	F: CGATGTGCAGTACCAGTAA<br>R: TTAGTGACCAGAATCAGCGG	585	60	B	[60]
bla$_{SHV}$	F: TTATCTCCCTGTTAGCCACC<br>R: GATTTGCTGATTTCGCTCGG	795	50	B	[61]
bla$_{OXA-1}$	F: ATATCTCTACTGTTGCATCTCC<br>R: AAACCCTTCAAACCATCC	619	54	B	[62]
	PMQR Genes				
*qnr*A	F: ATTTCTCACGCCAGGATTTG<br>R: GATCGGCAAAGGTTAGGTCA	516	54	B	[63]
*qnr*B	F: GATCGTGAAAGCCAGAAAGG<br>R: ACGATGCCTGGTAGTTGTCC	469	54	B	
*qnr*S	F: ACGACATTCGTCAACTGCAA<br>R: TAAATTGGCACCCTGTAGGC	417	54	B	
*qep*A	F: GCAGGTCCAGCAGCGGGTAG<br>R: CTTCCTGCCCGAGTATCGTG	199	60	B	[64]
	Carbapenemase Genes				
bla$_{IMP-1}$	F: TCTCATTTTCATAGRGACAG<br>R: ACCAGTTTTGCCTTACCATA	353	51	C	[15]
bla$_{KPC-gp}$	F: GCGGAACCATTCGCTAAACTC<br>R: CGCCCAACTCCTTCAGCAACA	340	55	C	
bla$_{NDM-1}$	F: GGCGGAATGGCTCATCACGA<br>R: CGCAACACAGCCTGACTTTC	286	60	C	
bla$_{VIM}$	F: GATGGTGTTTGGTCGCATA	390	52	C	

	R: CGAATGCGCAGCACCAG				
bla_{OXA-48}	F: GCGTGGTTAAGGATGAACAC<br>R: CATCAAGTTCAACCCAACCG	438	52	C	
	Virulence Associated Genes				
*wab*G	F: CTCTGGTGCGGCAGAAGTAC<br>R: TGGCCGTCGACGATAAACTC	931	52	D	
*rmp*A	F: ACCCTTTACAGCCAAATTTTCTTGT<br>R：CTGGGCTACCTCTGCTTCATAT	468	52	D	
*mag*A	F: TGATAAGTGGCGGAGATTCTGA<br>R：TGATAAGTGGCGGAGATTCTGA	542	52	D	
*ent*B	F: ATTTCCTCAACTTCTGGGGC<br>R: AGCATCGGTGGCGGTGGTCA	371	60	D	
kfu	F: GAAGTGACGCTGTTTCTGGC<br>R: TTTCGTGTGGCCAGTGACTC	797	55	D	[19]
*iro*N	F: GAATGAAACTACCGCCCCCA<br>R: TGTGGAGTGGAGGCGAGATA	1033	54	D	
*iuc*A	F: GCTTATTTCTCCCCAACCC<br>R: TCAGCCCTTTAGCGACAAG	583	59	D	
*all*S	F: CTTCAGCAGATAAATGACGGGGTAG<br>R: GTGGGTAAACCGCCATATTTTCC	244	56	D	
	MLST Genes				
*gap*A	F: TGAAATATGACTCCACTCACGG<br>R: CTTCAGAAGCGGCTTTGATGGCTT	662	60	E	
*inf*B	F: CTCGCTGCTGGACTATATTCG<br>R: CGCTTTCAGCTCAAGAACTTC	462	50	E	
mdh	F: CCCAACTCGCTTCAGGTTCAG<br>R: CCGTTTTTCCCCAGCAGCAG	756	50	E	
*pho*E	F: ACCTACCGCAACACCGACTTCTTCGG<br>R: TGATCAGAACTGGTAGGTGAT	602	50	E	[19]
pgi	F: GAGAAAAAACCTGCCTGTACTGCTGGC<br>R: CGCGCCACGCTTTATAGCGGTTAAT	566	50	E	
*rpo*B	F: GGCGAAATGGCWGAGAACCA<br>R: GAGTCTTCGAAGTTGTAACC	1075	50	E	
*ton*B	F: CTTTATACCTCGGTACATCAGGTT<br>R: ATTCGCCGGCTGRGCRGAGAG	539	45	E	

PCR profile A: Initial denaturation at 94 °C for 5 min; 30 cycles of denaturation at 94 °C for 1 min, annealing at the indicated temperature for 1 min, and extension at 72 °C for 1 min; final extension at 72 °C for 10 min.

PCR profile B: Initial denaturation at 94 °C for 3 min; 30 cycles of denaturation at 94 °C for 60 s, annealing at the target-specific temperature indicated in the table for 60 s, and extension at 72 °C for 60 s; final extension at 72 °C for 7 min.

PCR profile C: Initial denaturation at 94 °C for 3 min; 30 cycles of denaturation at 94 °C for 60 s, annealing at the target-specific temperature indicated in the table for 60 s, and extension at 72 °C for 60 s; final extension at 72 °C for 7 min.

PCR profile D: Initial denaturation at 95 °C for 3 min; 30 cycles of denaturation at 94 °C for 25 s, annealing at the target-specific temperature indicated in Table S2 for 25 s, and extension at 72 °C for 1 min; final extension at 72 °C for 5 min.

PCR profile E: Initial denaturation at 95 °C for 3 min; 30 cycles of denaturation at 94 °C for 25 s, annealing at the target-specific temperature indicated in Table S2 for 25 s, and extension at 72 °C for 1 min; final extension at 72 °C for 5 min.

Table S3. Molecular characterization, antimicrobial resistance, and virulence associated gene profiles of poultry origin KpSC isolates.

Isolate ID	Poultry origin	Species	16S rRNA gene accession number	Sequence type	Phenotypic antimicrobial resistance	Antimicrobial resistance determinants	No. of Resistant antimicrobial classes	MAR Index	Virulence associated genes
**16**	chicken	*K. pneumoniae*	PZ395315	5590	ampicillin, amoxicillin, enrofloxacin, trimethoprim/ sulfamethoxazole	*intI*1, bla_{TEM}	3	0.33	*wab*G, *ent*B
**42**	chicken	*K. pneumoniae*	PZ395316	5626	ampicillin, amoxicillin, enrofloxacin	*intI*1, bla_{SHV}	2	0.25	*wab*G, *ent*B
**43**	chicken	*K. quasipneumoniae*	PZ395317	9812	ampicillin, amoxicillin, enrofloxacin	*intI*1, $bla_{CTX\text{-}M}$	2	0.25	*wab*G, *ent*B
**58**	chicken	*K. pneumoniae*	PZ395318	292	ampicillin, amoxicillin, enrofloxacin	bla_{SHV}	2	0.25	*wab*G, *ent*B, *kfu*
**60**	chicken	*K. variicola*	PZ395319	6479	ampicillin, amoxicillin, enrofloxacin	bla_{SHV}, *qnr*B	2	0.25	*wab*G, *ent*B
**68***	turkey	*K. pneumoniae*	PZ395320	15	ESBL, ampicillin, amoxicillin, enrofloxacin, nalidixic acid, gentamicin, trimethoprim/ sulfamethoxazole, doxycycline, tetracycline	*intI*1, bla_{TEM}, $bla_{CTX\text{-}M}$, bla_{SHV}, *qnr*B, *qnr*S	5	0.67	*wab*G, *ent*B

*Isolate 68 exhibited an ESBL phenotype in the combined disk test (CDT).

Table S4. Isolate tracking and identification workflow from presumptive mucoid Enterobacterales isolates to the final sequence confirmed KpSC dataset.

**Identification stage**	**Isolate code**	**Identification result**	**Final disposition**
Direct 16S rRNA and *rpo*B sequencing	16, 42, 58	*K. pneumoniae*	Included as unique sequence confirmed KpSC isolates
Direct 16S rRNA and *rpo*B sequencing	43	*K. quasipneumoniae*	Included as a unique sequence confirmed KpSC isolate
Direct sequencing / isolate tracking	44	Same isolate as isolate 43	Excluded as a laboratory duplicate
Direct 16S rRNA and *rpo*B sequencing	60	*K. variicola*	Included as a unique sequence confirmed KpSC isolate
Direct 16S rRNA and *rpo*B sequencing	Remaining 4 isolates of the initial 10 presumptive isolates	Not confirmed as KpSC	Excluded
MALDI-TOF MS screening	53 of the remaining 58 presumptive isolates	Not identified as potential KpSC	Excluded from further sequence confirmation
MALDI-TOF MS screening	34, 41, 51, 53, 68	Potential KpSC	Referred for sequence confirmation or isolate tracking
Isolate tracking	41	Same isolate as isolate 42	Excluded as a laboratory duplicate
16S rRNA and *rpo*B sequence confirmation	34, 51, 53	Not confirmed as KpSC	Excluded
16S rRNA and *rpo*B sequence confirmation	68	*K. pneumoniae*	Included as a unique sequence confirmed KpSC isolate
Final confirmed dataset	16, 42, 43, 58, 60, 68	Six unique sequence confirmed KpSC isolates	Included in subsequent characterization
